# Modulation of Casimir Force between Graphene-Covered Hyperbolic Materials

**DOI:** 10.3390/nano12132168

**Published:** 2022-06-23

**Authors:** Ge Song, Zhixiang Liu, Lingchun Jia, Cong Li, Yingli Chang

**Affiliations:** College of Information Technology, Shanghai Ocean University, Shanghai 201306, China; gsong@shou.edu.cn (G.S.); cli@shou.edu.cn (C.L.); ylchang@shou.edu.cn (Y.C.)

**Keywords:** Casimir force, graphene, hyperbolic material

## Abstract

A flexible method for modulating the Casimir force is proposed by combining graphene and hyperbolic materials (HMs). The proposed structure employs two candidates other than graphene. One is hexagonal boron nitride (hBN), a natural HM. The other is porous silicon carbide (SiC), which can be treated as an artificial HM by the effective medium theory. The Casimir force between graphene-covered hBN (porous SiC) bulks is presented at zero temperature. The results show that covering HM with graphene increases the Casimir force monotonically. Furthermore, the force can be modulated by varying the Fermi level, especially at large separation distances. The reflection coefficients are thoroughly investigated, and the enhancement is attributed to the interaction of surface plasmons (SPs) supported by graphene and hyperbolic phonon polaritons (HPhPs) supported by HMs. Moreover, the Casimir force can be controlled by the filling factor of porous SiC. The Casimir force can thus be modulated flexibly by designing desired artificial HMs and tuning the Fermi level. The proposed models have promising applications in practical detection and technological fields.

## 1. Introduction

The Casimir force is an intriguing macroscopic effect caused by the quantum fluctuations of electromagnetic fields. Casimir predicted the existence of attractive forces between two parallel perfectly conducting plates in 1948 [1]. Lifshitz then generalized a theory of forces between two semi-infinite dielectric parallel plates with dispersive and absorptive properties at any temperature [2]. The Casimir effect is still a hot topic with the development of microelectromechanical and nanoelectromechanical systems (MEMS and NEMS). Over the last two decades, special emphasis has been placed on theoretical understanding [3,4,5,6] and precise experimental measurements [7,8,9,10,11,12,13] of the Casimir effect. In the study of Casimir force, the primary geometric configuration is two parallel plates of natural materials separated by a vacuum gap. In general, the force is too weak for practical detection, so enhancing weak Casimir forces is critical. Furthermore, the force is usually attractive and dominates in the submicrometer regime, where irreversible adhesion of neighboring elements in MEMS and NEMS can occur [14,15,16]. As a result, modulating the Casimir force is both fundamental and technological [5,17]. The realization of repulsive force is related to the symmetry of electric and magnetic properties of the boundary materials [18,19]. Consequently, using special materials with controllable electromagnetic properties to modulate the Casimir force becomes an interesting topic [4]. Metamaterials, for example, as a type of artificial materials, have unusual electromagnetic properties that natural materials do not have, and are used in cloaking [20], vacuum induced transparency [21], and controlling the Casimir effect [22,23,24]. Furthermore, saturated ferrite materials [25,26] and topological insulators [27,28,29] are proposed to modulate the Casimir effect.

Graphene, a two-dimensional sheet of carbon atoms arranged in a hexagonal lattice, has piqued the curiosity of many scientists [30]. The linear dispersion relation near the Dirac point causes an extraordinary response to light [31]. In particular, graphene can support surface plasmons (SPs) in the terahertz to infrared frequency ranges [32]. There has also been extensive research into using graphene to modulate the Casimir effect [32,33,34,35,36,37,38,39,40]. The plasmonic response of graphene is well understood to be highly dependent on the deposited substrate [41]. As a result, Goos-Hänchen shift [42,43], quantum interference [44], and Casimir friction [32,33] have been investigated in graphene-based models. For example, using graphene-covered hyperbolic materials (HMs) can significantly increase Casimir friction due to the coupling of SPs with hyperbolic phonon polaritons (HPhPs) supported by HMs [33]. This enhancement is active, because graphene’s optical conductivity is adjustable and can be controlled by an external field or gate voltage.

Hyperbolic materials have gotten a lot of attention in the last decade because of their unique electromagnetic properties [45]. Diagonal elements of a uniaxial HM’s permittivity tensor have opposite signs, resulting in a hyperbolic isofrequency contour for TM polarization [46]. It is possible to achieve ultrahigh propagating wave vectors and surface wave excitation by using HMs. Hexagonal boron nitride (hBN) is a natural HM with hyperbolic responses in the infrared frequency range [47]. Hyperbolic phonon polaritons can be supported by hBN, which has been thoroughly investigated [48], and as microfabrication technology develops, artificial HMs with hyperbolic responses in specific frequency bands can be constructed. In general, alternative metal-dielectric layered structures [49] or a lattice of nanowires embedded in a dielectric matrix [46] can be used to realize artificial HMs. Ingredient materials and their proportions can be used to control the desired electromagnetic properties. As a result, when compared to natural HMs, artificial HMs provide additional methods for modulating HPhPs.

It is well known that the electromagnetic properties of the boundary material can modify the Casimir force [6]. In this paper, we investigate the Casimir force between graphene-covered HMs. The remainder of this paper is organized as follows. Section 2 introduces the two models under consideration here, as well as the Casimir force between two graphene-covered HMs. Section 3 demonstrates the modulation of Casimir force caused by the interaction of SPs and HPhPs. The results show that the Casimir force can be actively modulated by the Fermi level and artificial HMs. Section 4 is where we draw our conclusions.

## 2. Materials and Methods

**Figure 1:**
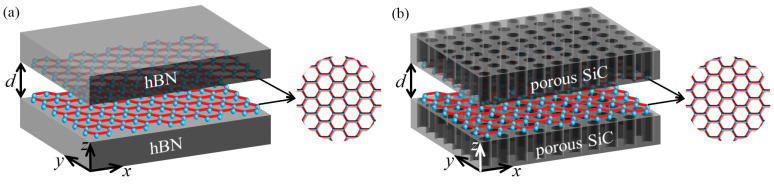
The Casimir force between two graphene-covered HMs separated by *d* is depicted schematically. Two models under consideration: (**a**) graphene-covered hBN and (**b**) graphene-covered porous SiC.

Figure 1 depicts the scheme that takes into account two different models. The Casimir force between two identical samples separated by *d* is investigated in each model. One model’s samples are graphene-covered hBN bulks, as shown in Figure 1a, while the other model’s samples are graphene-covered porous silicon carbide (SiC) bulks as shown in Figure 1b. The models are in free space, with the x−y plane parallel to the graphene plane. The optical properties of graphene in the low-frequency range and high doping limit are determined by its in-plane conductivity σ, which can be expressed as [42]

(1) $$\sigma \left(\omega \right)=\frac{ie^{2}E_{F}}{\pi \hbar^{2}\left(\omega +i\tau^{-1}\right)},$$

under the random phase approximation (RPA). Here, *e* is the electron charge, $E_{F}$ is the Fermi level, and $\tau =\mu E_{F}/ev_{F}^{2}$ is the relaxation time caused by electron doping, electron defect, and phonon scattering. The mobility of the graphene charge carriers is $\mu =10^{4}-10^{6}{\mathrm{cm}}^{2}\;V^{-1}s^{-1}$, and the Fermi velocity is $v_{F}=10^{6}\;m/s$.

**Figure 2:**
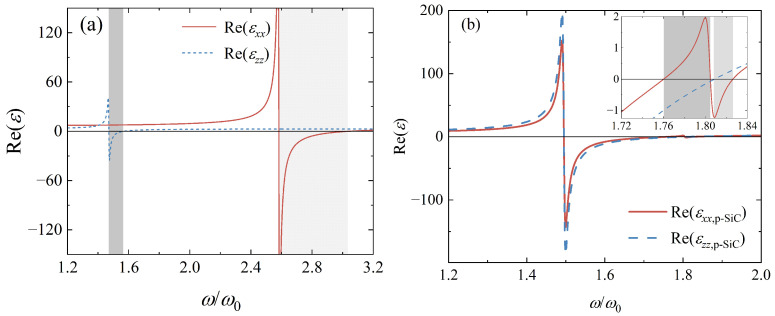
(**a**) The relationship between the real parts of $\varepsilon_{l,\mathrm{hBN}}$ and ω. (**b**) The relationship between the real parts of $\varepsilon_{l,p-\mathrm{SiC}}$ and ω with f=0.3. Here l=xx,zz and grey shadow zones indicate the hyperbolic bands. The inset shows the real parts of $\varepsilon_{l,p-\mathrm{SiC}}$ from $1.72\;\omega_{0}$ to $1.84\;\omega_{0}$.

The hBN is a naturally anisotropic material that exhibits hyperbolic dispersion. The permittivity of hBN is a tensor, and the elements of the anisotropic permittivity tensor are as follows [33]

(2) $$\varepsilon_{l,\mathrm{hBN}}=\varepsilon_{l,\infty }\left[1+\frac{\omega_{\mathrm{LO},l}^{2}-\omega_{\mathrm{TO},l}^{2}}{\omega_{\mathrm{TO},l}^{2}-\omega^{2}-i\omega \gamma_{l}}\right],$$

where l=xx,zz, LO and TO are two phonon modes, and γ is the damping coefficient. The parameters are $\varepsilon_{xx,\infty }=4.87$, $\omega_{\mathrm{LO},xx}$ = 3.0348 $\omega_{0}$, $\omega_{\mathrm{TO},xx}=2.5824\;\omega_{0}$, $\gamma_{xx}=0.0094\;\omega_{0}$, $\varepsilon_{zz,\infty }=2.95$, $\omega_{\mathrm{LO},zz}=1.5645\;\omega_{0}$, $\omega_{\mathrm{TO},zz}=1.4703\;\omega_{0}$, $\gamma_{zz}=0.0075\;\omega_{0}$, and $\omega_{0}=10^{14}\;\mathrm{rad}/s$. The real parts of $\varepsilon_{l,\mathrm{hBN}}$ are plotted as functions of ω in Figure 2a. Two grey shadow zones satisfying ReεxxReεzz<0 are obtained, in which HPhPs can be excited. Electromagnetic waves possess a high wave vector in such bands, thus the large electromagnetic local density of the state can be obtained [46].

Bulk SiC is an isotropic material, and its permittivity can be described by the Lorentz model [40]

(3) $$\varepsilon_{s}\left(\omega \right)=\varepsilon_{\infty }\frac{\omega^{2}-\omega_{L}^{2}+i\gamma \omega }{\omega^{2}-\omega_{T}^{2}+i\gamma \omega },$$

where $\varepsilon_{\infty }=6.7$, $\omega_{L}=1.827\;\omega_{0}$, $\omega_{T}=1.495\;\omega_{0}$, and $\gamma =0.009\;\omega_{0}$. The desired kind of anisotropy can be generated by the structure of a lattice of nanowires embedded in a dielectric matrix. Therefore, by embedding a lattice of air cylinders in a SiC, an artificial HM can be fabricated. By using the Maxwell-Garnett method [50], the effective permittivity of such porous SiC is described as

(4) $$\varepsilon_{xx,p-\mathrm{SiC}}=\varepsilon_{yy,p-\mathrm{SiC}}=\frac{\left[\left(1+f\right)+\left(1-f\right)\varepsilon_{s}\right]\varepsilon_{s}}{\left(1-f\right)+\left(1+f\right)\varepsilon_{s}},$$

(5) $$\varepsilon_{zz,p-\mathrm{SiC}}=f+\left(1-f\right)\varepsilon_{s},$$

where the filling factor *f* is the area percentage occupied by air holes in the xy section of the medium. The real parts of $\varepsilon_{l,p-\mathrm{SiC}}$ (l=xx,zz) as functions of ω with f=0.3 are presented in Figure 2b. Two hyperbolic bands of porous SiC are obtained, as shown by the inset in Figure 2b. Artificial materials similar to such porous SiC are also named hyperbolic metamaterials that can also support HPhPs.

By utilizing the Maxwell electromagnetic stress tensor method with the properties of macroscopic field operators, the Casimir force at zero temperature is eventually expressed as [23]

(6) F=−ℏπRe∫0∞dω∫∫d2k‖2πω2c2−k‖2∑p=TE,TMr1p(ω,k)r2p(ω,k)e2idω2/c2−k‖21−r1p(ω,k)r2p(ω,k)e2idω2/c2−k‖2,

where the integral is carried out over all electromagnetic modes. The wave vector component $k_{\|}$ is parallel to the x−y plane. The reflection coefficient from the space between two samples to the surface of top (bottom) sample for a *p* polarized wave is denoted by $r_{1p}$ ($r_{2p}$). Since the top and bottom samples in each model are identical, $r_{1p}$ equals $r_{2p}$. We shall omit the subscripts 1 and 2. All the singularities can be avoided by converting the integral of positive real ω to that of positive imaginary frequency ξ, i.e., ω=iξ, the Casimir force can be written as

(7) $$F=\frac{\hbar }{2\pi^{2}}\int_{0}^{\infty }d\xi \int_{0}^{\infty }k_{\|}dk_{\|}\sqrt{\frac{\xi^{2}}{c^{2}}+k_{\|}^{2}}\sum_{p=\mathrm{TE},\mathrm{TM}}\frac{r_{p}\left(i\xi ,k\right)r_{p}\left(i\xi ,k\right)e^{-2d\sqrt{\xi^{2}/c^{2}+k_{\|}^{2}}}}{1-r_{p}\left(i\xi ,k\right)r_{p}\left(i\xi ,k\right)e^{-2d\sqrt{\xi^{2}/c^{2}+k_{\|}^{2}}}}.$$

To compute the Casimir force, the reflection coefficients are obtained using the approach described in [51]. Graphene is a monolayer in this study that can be considered as a conductivity current. Appendix A contains the detailed derivation of $r_{\mathrm{TE}}$ and $r_{\mathrm{TM}}$. The reflection coefficient of the graphene-covered HM for the TE polarized wave can be written as

(8) $$r_{\mathrm{TE}}=\frac{k_{iz}-k_{tz}^{\mathrm{TE}}-\sigma \omega \mu_{0}}{k_{iz}+k_{tz}^{\mathrm{TE}}+\sigma \omega \mu_{0}},$$

where $k_{iz}=\sqrt{k_{0}^{2}-k_{\|}^{2}}$ and $k_{tz}^{\mathrm{TE}}=\sqrt{\varepsilon_{xx}k_{0}^{2}-k_{\|}^{2}}$. The wave vector in free space is $k_{0}=\omega /c$. Since only $\varepsilon_{xx}$ appears in $r_{\mathrm{TE}}$, HPhPs can not be excited by TE polarized waves. The reflection coefficient for the TM polarized wave is expressed as

(9) $$r_{\mathrm{TM}}=\frac{\varepsilon_{xx}k_{iz}-k_{tz}^{\mathrm{TM}}+\frac{\sigma k_{iz}k_{tz}^{\mathrm{TM}}}{\omega \varepsilon_{0}}}{\varepsilon_{xx}k_{iz}+k_{tz}^{\mathrm{TM}}+\frac{\sigma k_{iz}k_{tz}^{\mathrm{TM}}}{\omega \varepsilon_{0}}},$$

where $k_{tz}^{\mathrm{TM}}=\sqrt{\varepsilon_{xx}k_{0}^{2}-k_{\|}^{2}\varepsilon_{xx}/\varepsilon_{zz}}$. Obviously, TM polarized waves can excite HPhPs since both $\varepsilon_{xx}$ and $\varepsilon_{zz}$ appear in $r_{\mathrm{TM}}$. Furthermore, the reflection coefficients are affected by graphene conductivity, implying that SPs supported by graphene can couple with electromagnetic modes supported by HM, particularly HPhPs. Because the optical properties of the sample can be conveniently turned by varying $E_{F}$, the Casimir force, which is usually dependent on the surrounding environment, can be controlled by $E_{F}$.

## 3. Results and Discussion

### 3.1. Casimir Force of Graphene-Covered hBN

**Figure 3:**
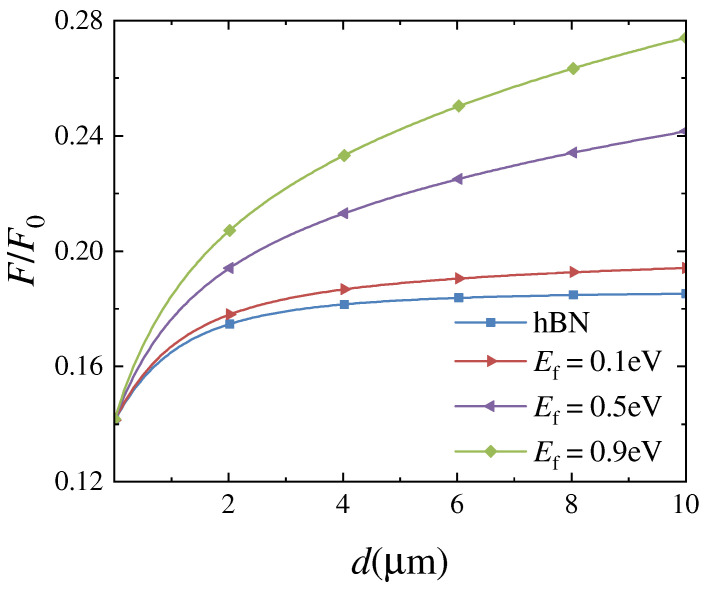
The relationship between the relative Casimir force and the separating distance *d*. The blue line represents the force between two identical hBN bulks, while the other lines represent the force between two graphene-covered hBN bulks at different Fermi levels.

The relative Casimir forces between two identical graphene-covered hBN bulks as a function of separating distance *d* for various Fermi levels are presented in Figure 3. The Casimir force is scaled by the well-known formula $F_{0}=\hbar c\pi^{2}/240d^{4}$, which is the Casimir force per unit area between two parallel perfectly conducting plates separated by *d*. Because two samples have the same electric and magnetic properties, the force is obviously attractive at any distance. The relative force between two identical hBN bulks is also plotted for comparison purposes, as shown by the blue line in Figure 3. The relative force is clearly increased when hBN bulks are covered by graphene. In addition, as the Fermi level $E_{f}$ increases, the relative force increases monotonically for arbitrary separating distances. The relative force is insensitive to $E_{f}$ for minimal *d*, and curve slopes are large. However, for large *d*, the relative force is sensitive to $E_{f}$, but curve slopes are small. Since the Fermi level is adjustable, the Casimir force can be controlled flexibly.

**Figure 4:**
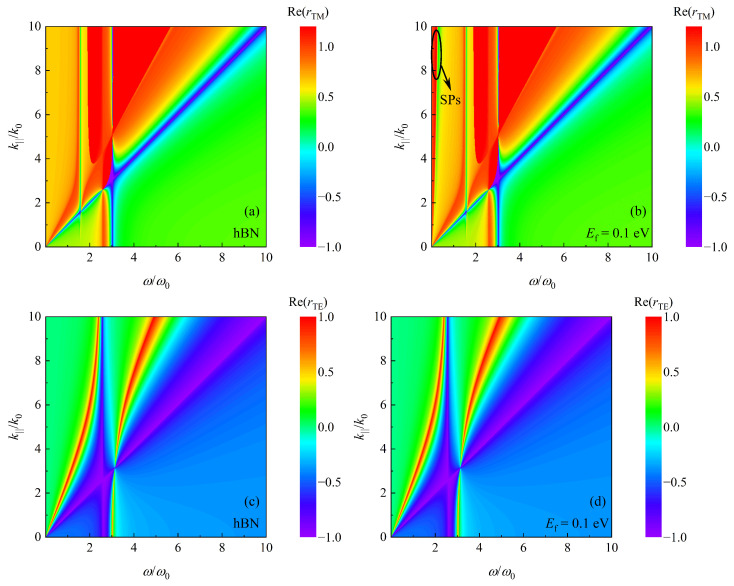
Re(rTM) or Re(rTE) as function of ω and $k_{\left|\right|}$. (**a**) Re(rTM) of hBN. (**b**) Re(rTM) of graphene-covered hBN with Fermi level $E_{f}=0.1\;\mathrm{eV}$. (**c**) Re(rTE) of hBN. (**d**) Re(rTE) of graphene-covered hBN with Fermi level $E_{f}=0.1\;\mathrm{eV}$.

The Casimir force is related to all electromagnetic modes supported by two samples. From Equation (6), we know that electromagnetic modes are represented by the reflection coefficients of samples. Thus, both real parts of $r_{\mathrm{TM}}$ and $r_{\mathrm{TE}}$ are plotted as a function of frequency ω and wave vector component $k_{\left|\right|}$ in Figure 4. Comparing Re(rTM) of hBN and Re(rTM) of graphene-covered hBN, i.e., Figure 4a,b, reflection coefficients are enhanced clearly when $\omega <0.1\;\omega_{0}$ for all $k_{\left|\right|}$. However, it is difficult to distinguish between Re(rTE) of hBN and Re(rTE) of graphene-covered hBN by comparing Figure 4c,d. According to Equation (8), when hBN is covered by graphene, $r_{\mathrm{TE}}$ is affected by σ beyond $\varepsilon_{xx,\mathrm{hBN}}$. By analyzing Equation (9), when the sample is graphene-covered hBN, $r_{\mathrm{TM}}$ is affected by $\varepsilon_{xx,\mathrm{hBN}}$, $\varepsilon_{zz,\mathrm{hBN}}$ and σ, whereas it only relates to the permittivity of hBN when the graphene is absent. Therefore, SPs supported by graphene are mainly coupled with HPhPs supported by hBN. As a result, the Casimir force is enhanced by covering hBN with graphene, as shown in Figure 3. Therefore, this study focuses on the coupling of SPs and HPhPs excited by a TM polarized wave.

**Figure 5:**
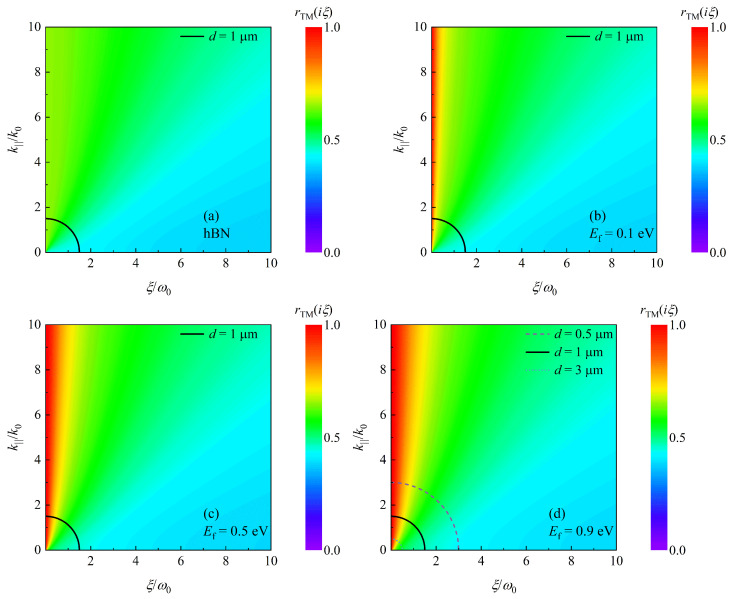
$r_{\mathrm{TM}}\left(i\xi \right)$ as function of ξ and $k_{\left|\right|}$. (**a**) is the case of hBN. (**b**–**d**) are the cases of graphene-covered hBN with Fermi levels (**b**) $E_{f}=0.1\;\mathrm{eV}$, (**c**) $E_{f}=0.5\;\mathrm{eV}$ and (**d**) $E_{f}=0.9\;\mathrm{eV}$, respectively. Solid curves in panels (**a**–**d**) indicate the arc of a circle $\xi^{2}/c^{2}+k_{\left|\right|}^{2}={\left(1/2d\right)}^{2}$ for d=1μm. Dashed and dotted lines in panel (**d**) indicate the cases for d=0.5μm and d=3μm, respectively.

To extract the contribution of SPs and HPhPs to the enhancement of the Casimir force, the TM reflection coefficients as a function of imaginary frequency ξ and wave vector component $k_{\left|\right|}$ are presented in Figure 5. When comparing Figure 5a,b, it is clear that $r_{\mathrm{TM}}\left(i\xi \right)$ is enhanced at low frequencies when the hBN is covered by graphene, and as shown in Figure 5b–d, the enhancement area grows as the Fermi level increases. In Equation (7), the term $\exp(-2d\sqrt{\xi^{2}/c^{2}+k_{\left|\right|}^{2}})$ acts as a truncated function. The arc of a circle $\xi^{2}/c^{2}+k_{\left|\right|}^{2}={\left(1/2d\right)}^{2}$ for d=1 μm is plotted in Figure 5. The amplitude of the force can be represented by reflection coefficients inside the arc [23]. Clearly, the proportion of high reflection coefficients inside this arc grows as $E_{F}$ increases. Correspondingly, the Casimir force is getting larger for d=1 μm with increasing $E_{F}$, as shown in Figure 3. Furthermore, the radius of a circular arc is inversely proportional to the separating distance *d*. In Figure 5d, we also plot the curves for d=0.5 μm and d=3 μm. Obviously, with increasing *d*, i.e., decreasing radius, the proportion of high reflection coefficients is increasing. As a result, the Casimir force tends to $F_{0}$ with expanding *d*, as illustrated in Figure 3.

### 3.2. Casimir Force of Graphene-Covered Porous SiC

**Figure 6:**
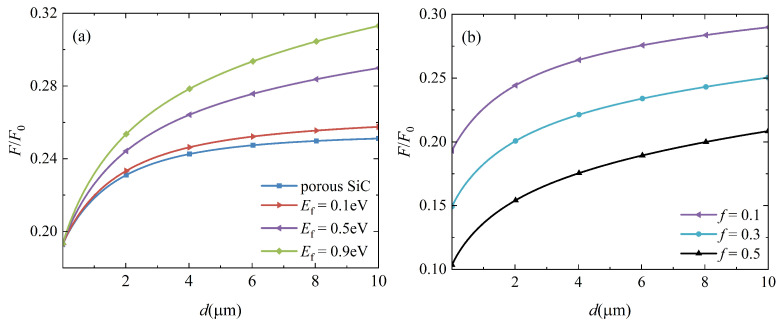
Dependence of the Casimir force on the separation *d*. (**a**) The case of identical porous SiC is indicated by the blue line, while other lines represent the case of graphene-covered porous SiC at different Fermi levels. The filling factor has been set to f=0.1. (**b**) The case of graphene-covered porous SiC for different filling factors with a fixed Fermi level $E_{f}=0.5\;\mathrm{eV}$.

The Casimir force between two identical graphene-covered porous SiC bulks is also investigated in relation to the separating distance *d*. The filling factor is f=0.1, and the forces for varying Fermi levels are presented in Figure 6a. The force between two porous SiC bulks is plotted for comparison purposes, as shown by the blue line in Figure 6a. Similar to the case of graphene-covered hBN, the Casimir force increases monotonically with increasing Fermi levels, particularly at large separating distances. These results are expected according to the above analysis because porous SiC is also an HM. Furthermore, when the Fermi level and separating distance are fixed, a more significant force can be obtained in the graphene-covered porous SiC configuration than in the graphene-covered hBN bulks, as shown in Figure 3. As shown in Figure 2b, both $\varepsilon_{xx,p-\mathrm{SiC}}$ and $\varepsilon_{zz,p-\mathrm{SiC}}$ are negative in the range 1.496 $\omega_{0}$ to 1.761 $\omega_{0}$, indicating that porous SiC also excites electromagnetic modes other than HPhPs. These modes are all coupled and contribute to the Casimir force.

**Figure 7:**
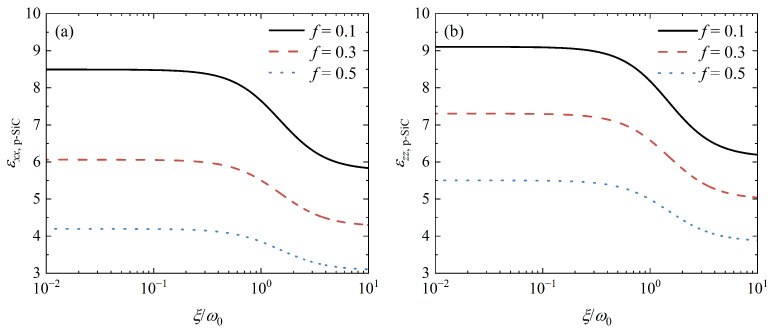
(**a**) $\varepsilon_{xx,p-\mathrm{SiC}}$ and (**b**) $\varepsilon_{zz,p-\mathrm{SiC}}$ as a function of ξ for different filling factors *f*.

As an artificial HM, the permittivity of porous SiC can be modulated by the filling factor *f*. Therefore, *f* influences the coupling of SPs supported by graphene and HPhPs supported by porous SiC, which can be used to control the Casimir force. Figure 6b depicts the Casimir force as a function of the separating distance *d* for different filling factors. When *f* increases, the relative force for arbitrary separations decreases dramatically. This outcome is simple to comprehend. As *f* increases, so does the proportion of air holes. According to Equations (4) and (5), the electric properties of porous SiC should decrease as *f* increases. As a result, the Casimir force decreases as the contribution of these modes supported by porous SiC decreases. Figure 7 depicts the permittivity of porous SiC as a function of ξ for different filling factors to demonstrate this explanation. As the filling factor *f* increases, for arbitrary ξ, both $\varepsilon_{xx,p-\mathrm{SiC}}$ and $\varepsilon_{zz,p-\mathrm{SiC}}$ decrease, confirming our prediction. Furthermore, when $\xi <\omega_{0}$, all curves in Figure 7 are almost flat, but sloping when $\omega_{0}<\xi <10\;\omega_{0}$. As previously stated, the force at a small separation distance is primarily derived from electromagnetic modes in the high-frequency region, whereas modes in the low-frequency region mainly contribute to the force at a large separation distance [22]. As shown in Figure 6b, slopes of relative forces at a small separation distance are large, while slopes at a large separation distance are small.

### 3.3. Discussion

The Casimir force per unit area between two parallel graphene sheets is inversely proportional to $d^{4}$ at zero temperature, but with a substantially smaller coefficient when compared with that of two perfectly conducting plates [38]. According to the data in [38], the Casimir force per $1\;{\mathrm{cm}}^{2}$ is around $0.006\;F_{0}=7.8\times 10^{-6}\;N$ when d=0.1μm. For the same area and *d*, when $E_{f}=0.1\;\mathrm{eV}$, *F* is around $0.145\;F_{0}=1.9\times 10^{-4}\;N$ in Figure 3, and $0.197\;F_{0}=2.6\times 10^{-4}\;N$ in Figure 6a. Therefore, mounting graphene on an HM substrate increases the Casimir force by more than one order of magnitude. Furthermore, the Casimir force per $1\;{\mathrm{cm}}^{2}$ between two identical artificial HMs is around $0.1\;F_{0}=8.1\times 10^{-10}\;N$ at d ≈ 2μm [23]. To compare, when $E_{f}=0.1\;\mathrm{eV}$, *F* is about $0.178\;F_{0}=1.5\times 10^{-9}\;N$ in Figure 3, and $0.233\;F_{0}=1.9\times 10^{-9}\;N$ in Figure 6a. Covering an HM with graphene monolayer therefore enhances the Casimir force.

Since the samples in this study are in free space, the detection system should be set in a high-vacuum chamber to measure the Casimir force between graphene-covered HMs. Furthermore, the quality of the graphene layer should be checked to guarantee that it is a monolayer. The gradient of the Casimir force may be measured using the experimental setup described in [7]. However, there are some problems. One difficulty is that maintaining two flat samples parallel over small distances is quite challenging. Most experiments chose one of the samples to be spherical with a large radius. As a result, the geometry will influence the magnitude of the Casimir force. Another issue is the effect of finite temperature since the temperature considered here is zero. The Casimir force at finite temperature can be obtained by substituting the integration along with the imaginary frequency ξ axis in Equation (7) with the summation over the Matsubara frequencies [24]. The influences of geometry and finite temperature on the Casimir force of our proposal are yet to be investigated further.

## 4. Conclusions

In conclusion, we investigate the Casimir force between two identical graphene-covered HMs. The first model’s samples are graphene-covered hBN bulks. When graphene covers hBN, the Casimir force increases for arbitrary separation distances, and as the Fermi level $E_{f}$ increases, the force increases monotonically. The reflection coefficients of samples are thoroughly examined. SPs supported by graphene and HPhPs supported by hBN are coupled, and these electromagnetic modes are related to the enhancement of the Casimir force. Furthermore, the hBN is replaced by porous SiC, which is treated as an artificial HM following the effective medium theory. When $E_{f}$ increases, the Casimir force is still increased monotonically. However, as the filling factor *f* increases, the force decreases for arbitrary separation distances. The electromagnetic responses of porous SiC are used to understand this phenomenon. As a result, by designing suitable artificial HMs and tuning the Fermi level $E_{f}$, the desired Casimir force between graphene-covered HMs can be controlled. By combining graphene and HM, this work provides a flexible way to modulate the Casimir effect, which can use for the detection of Casimir force.

## Figures and Tables

**Figure A1:**
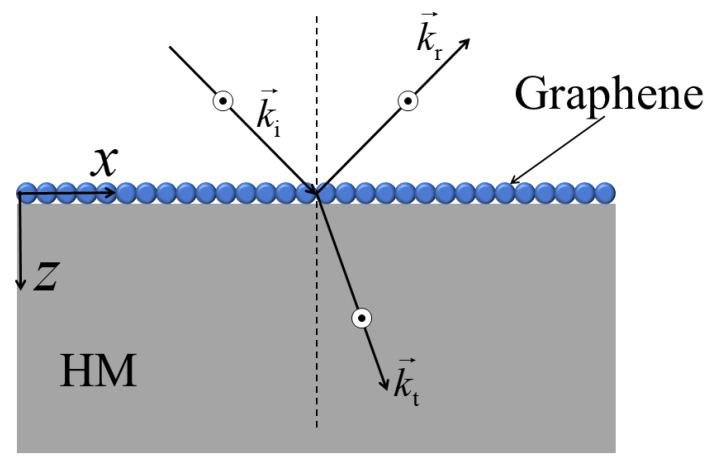
Schematic of electromagnetic wave scattering at the interface of vacuum and a graphene-covered HM.

## Data Availability

The data presented in this study are available on request from the corresponding author.

## Appendix A

Figure A1 depicts a schematic of electromagnetic wave scattering at the interface of vacuum and a graphene-covered HM. Assuming graphene is located in z=0. The medium in z<0 is vacuum, whereas the HM occupies z>0 space. An electromagnetic wave incident from the vacuum into the graphene-covered HM produces a reflected field in the vacuum and a transmitted field in the HM. Because graphene is a monolayer, it can be treated as a conducting sheet overlaying on an HM with infinite thickness in this work. We consider the TM polarized wave, and the magnetic fields of the incident, reflected, and transmitted waves can be written as

(A1) $${\vec{H}}_{i}=Ae^{i[\left(k_{\left|\right|}x+k_{iz}z\right)-\omega t]}\vec{j}$$

(A2) $${\vec{H}}_{r}=Re^{i[\left(k_{\left|\right|}x-k_{iz}z\right)-\omega t]}\vec{j}$$

(A3) $${\vec{H}}_{t}=Te^{i[\left(k_{\left|\right|}x+k_{tz}^{\mathrm{TM}}z\right)-\omega t]}\vec{j}$$

where *A*, *R*, and *T* denote the magnetic field magnitudes of the incident, reflected, and transmitted fields, respectively. The electric fields of the incident, reflected, and transmitted waves can be expressed using Maxwell’s equations as

(A4) $${\vec{E}}_{i}=\frac{A}{\omega \varepsilon_{0}}e^{i[\left(k_{\left|\right|}x+k_{iz}z\right)-\omega t]}\left(k_{iz}\vec{i}-k_{\left|\right|}\vec{k}\right)$$

(A5) $${\vec{E}}_{r}=\frac{R}{\omega \varepsilon_{0}}e^{i[\left(k_{\left|\right|}x-k_{iz}z\right)-\omega t]}\left(-k_{iz}\vec{i}-k_{\left|\right|}\vec{k}\right)$$

(A6) $${\vec{E}}_{t}=\frac{T}{\omega \varepsilon_{0}}e^{i[\left(k_{\left|\right|}x+k_{tz}^{\mathrm{TM}}z\right)-\omega t]}\left(\frac{k_{tz}^{\mathrm{TM}}}{\varepsilon_{xx}}\vec{i}-\frac{k_{\left|\right|}}{\varepsilon_{zz}}\vec{k}\right)$$

where $\varepsilon_{xx}$ and $\varepsilon_{zz}$ are diagonal elements of the permittivity tensor of a uniaxial HM. $k_{\left|\right|}^{2}+k_{iz}^{2}=k_{0}^{2}$ and $k_{\left|\right|}^{2}/\varepsilon_{zz}+{\left(k_{tz}^{\mathrm{TM}}\right)}^{2}/\varepsilon_{xx}=k_{0}^{2}$ are satisfied. Since the graphene layer is treated as a conducting sheet, the boundary conditions are expressed as

(A7) $$\vec{k}\times \left[{\vec{E}}_{t}-\left({\vec{E}}_{i}+{\vec{E}}_{r}\right)\right]=0$$

(A8) $$\vec{k}\times \left[{\vec{H}}_{t}-\left({\vec{H}}_{i}+{\vec{H}}_{r}\right)\right]=\vec{J}$$

where $\vec{J}$ is the conductivity current. The current $\vec{J}$ contains just the *x* component since the electric field $\vec{E}$ is in the xz plane. By inserting Equations (A1)–(A6) into Equations (A7) and (A8), we get

(A9) $$\frac{T}{\omega \varepsilon_{0}}\frac{k_{tz}^{\mathrm{TM}}}{\varepsilon_{xx}}=\frac{A}{\omega \varepsilon_{0}}\frac{k_{iz}}{\varepsilon_{1}}-\frac{R}{\omega \varepsilon_{0}}\frac{k_{iz}}{\varepsilon_{1}}$$

(A10) $$-\left[T-\left(A+R\right)\right]=\sigma \frac{T}{\omega \varepsilon_{0}}\frac{k_{tz}^{\mathrm{TM}}}{\varepsilon_{xx}}$$

The reflection coefficient for the TM polarized wave can be derived and expressed as

(A11) $$r_{\mathrm{TM}}=\frac{R}{A}=\frac{\varepsilon_{xx}k_{iz}-k_{tz}^{\mathrm{TM}}+\frac{\sigma k_{iz}k_{tz}^{\mathrm{TM}}}{\omega \varepsilon_{0}}}{\varepsilon_{xx}k_{iz}+k_{tz}^{\mathrm{TM}}+\frac{\sigma k_{iz}k_{tz}^{\mathrm{TM}}}{\omega \varepsilon_{0}}}$$

If the incident wave is TE polarized, the incident, reflected, and transmitted electric fields can be represented as

(A12) $${\vec{E}}_{i}=Ae^{i[\left(k_{\left|\right|}x+k_{iz}z\right)-\omega t]}\vec{j}$$

(A13) $${\vec{E}}_{r}=Re^{i[\left(k_{\left|\right|}x-k_{iz}z\right)-\omega t]}\vec{j}$$

(A14) $${\vec{E}}_{t}=Te^{i[\left(k_{\left|\right|}x+k_{tz}^{\mathrm{TE}}z\right)-\omega t]}\vec{j}$$

where *A*, *R*, and *T* represent the electric field magnitudes of the incident, reflected, and transmitted waves, respectively. $k_{\left|\right|}^{2}+{\left(k_{tz}^{\mathrm{TE}}\right)}^{2}=\varepsilon_{xx}k_{0}^{2}$ is satisfied. The magnetic fields can then be expressed as

(A15) $${\vec{H}}_{i}=\frac{A}{\omega \mu_{0}}e^{i[\left(k_{\left|\right|}x+k_{iz}z\right)-\omega t]}\left(-k_{iz}\vec{i}+k_{\left|\right|}\vec{k}\right)$$

(A16) $${\vec{H}}_{r}=\frac{R}{\omega \mu_{0}}e^{i[\left(k_{\left|\right|}x-k_{iz}z\right)-\omega t]}\left(k_{iz}\vec{i}+k_{\left|\right|}\vec{k}\right)$$

(A17) $${\vec{H}}_{t}=\frac{T}{\omega \mu_{0}}e^{i[\left(k_{\left|\right|}x+k_{tz}^{\mathrm{TE}}z\right)-\omega t]}\left(-k_{tz}^{\mathrm{TE}}\vec{i}+k_{\left|\right|}\vec{k}\right)$$

Since we are considering a TE polarized wave, the current $\vec{J}$ has just the *y* component. Substituting Equations (A12)–(A17) into Equations (A7) and (A8), we get

(A18) T−(A+R)=0

(A19) $$-\frac{T}{\omega \mu_{0}}k_{tz}^{\mathrm{TE}}-\left(-\frac{A}{\omega \mu_{0}}k_{iz}+\frac{R}{\omega \mu_{0}}k_{iz}\right)=\sigma T$$

The reflection coefficient for the TE polarized wave can be derived and expressed as

(A20) $$r_{\mathrm{TE}}=\frac{R}{A}=\frac{k_{iz}-k_{tz}^{\mathrm{TE}}-\sigma \omega \mu_{0}}{k_{iz}+k_{tz}^{\mathrm{TE}}+\sigma \omega \mu_{0}}$$
